# Progress in Photochemical and Electrochemical C–N Bond Formation for Urea Synthesis

**DOI:** 10.1021/acs.accounts.3c00424

**Published:** 2023-10-19

**Authors:** Hakhyeon Song, Danae A. Chipoco Haro, Po-Wei Huang, Luisa Barrera, Marta C. Hatzell

**Affiliations:** † George W. Woodruff School of Mechanical Engineering, Georgia Institute of Technology, Atlanta, Georgia 30332, United States; ‡ School of Materials Science and Engineering, Georgia Institute of Technology, Atlanta, Georgia 30332, United States; § School of Chemical and Biomolecular Engineering, Georgia Institute of Technology, Atlanta, Georgia 30332, United States

## Abstract

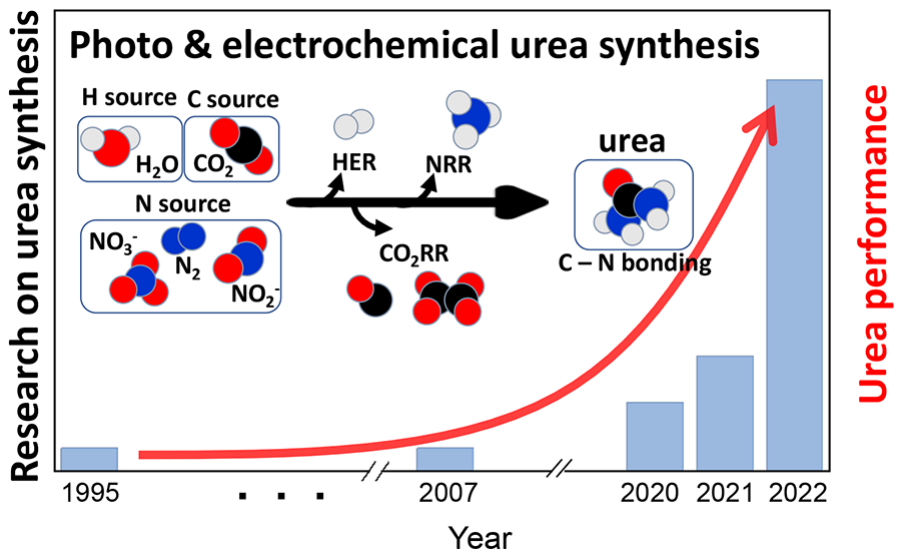

Here, we discuss recent advances and pressing challenges in achieving sustainable urea synthesis. Urea stands out as the most prevalent nitrogen-based fertilizer used across the globe, making up over 50% of all manufactured fertilizers. Historically, the Bosch-Meiser process has been the go-to chemical manufacturing method for urea production. This procedure, characterized by its high-temperature and high-pressure conditions, reacts ammonia with carbon dioxide to form ammonium carbamate. Subsequently, this ammonium carbamate undergoes dehydration, facilitated by heat, producing solid urea. A concerning aspect of this method is its dependency on fossil fuels, as nearly all the process heat comes from nonrenewable sources. Consequently, the Bosch-Meiser process leaves behind a considerable carbon footprint. Current estimates predict that unchecked, carbon emissions from urea production alone might skyrocket, reaching a staggering 286 Mt_CO2,eq_/yr by 2050. Such projections paint a clear picture regarding the necessity for more eco-friendly, sustainable urea production methods. Recently, the scientific community has shown growing interest in forming C–N bonds using alternative methods. Shifting toward photochemical or electrochemical processes, as opposed to traditional thermal-based processes, promises the potential for complete electrification of urea synthesis. This shift toward process electrification is not just an incremental change; it represents a groundbreaking advancement, the first of many steps, toward achieving deep decarbonization in the chemical manufacturing sector. Since the turn of 2020, there has been a surge in research focusing on photochemical and electrochemical urea synthesis. These methods capitalize on co-reduction of carbon dioxide with nitrogenous reactants like NOx and N_2_. Despite the progress, there are significant challenges that hinder these processes from reaching their full potential. In this comprehensive review, we shed light on the advances made in electrified C–N bond formation. More importantly, we focus on the invaluable insights gathered over the years, especially concerning catalytic reaction mechanisms. We have dedicated a section to underline key focal areas for up-and-coming research, emphasizing catalyst, electrolyte, and reactor design. It is undeniable that catalyst design remains at the heart of the matter, as managing the co-reduction of two distinct reactants (CO_2_ and nitrogenous species) is complex. This process results in a myriad of intermediates, which must be adeptly managed to both maintain catalyst activity and avoid catalyst deactivation. Moreover, the electrolytes play a pivotal role, essentially dictating the creation of optimal microenvironments that drive reaction selectivity. Finally, reactor engineering stands out as crucial to ensure optimal mass transport for all involved reactants and subsequent products. We touch upon the broader environmental ramifications of urea production and bring to light potential obstacles for alternative synthesis routes. A notable mention is the urgency of accelerating the uptake and large-scale implementation of renewable energy sources.

## Key References

- ComerB. M.; LiuY.-H.; DixitM. B.; HatzellK. B.; YeY.; CrumlinE. J.; HatzellM. C.; MedfordA. J.The role of adventitious carbon in photocatalytic nitrogen fixation by titania. J. Am. Chem. Soc.. 2018, 140, 15157–15160.3037205510.1021/jacs.8b08464(1) Surface carbon is crucial for photon-driven N_2_ and TiO_2_ interaction on titania, indicating a carbon-assisted nitrogen reduction mechanism.
- LimJ.; LiuC.-Y.; ParkJ.; LiuY.-H.; SenftleT. P.; LeeS. W.; HatzellM. C.Structure sensitivity of Pd facets for enhanced electrochemical nitrate reduction to ammonia. ACS Catal.2021, 11, 7568–7577.(2) Exploring Pd surface structure’s impact on nitrate and nitrite reduction, the study uses nanocatalysts with specific facets. Results highlight Pd(111) and Pd(100) facets in nitrate and nitrite reduction, with cuboctahedron most efficient for ammonia production.
- LimJ.; ChenY.; CullenD. A.; LeeS. W.; SenftleT. P.; HatzellM. C.PdCu electrocatalysts for selective nitrate and nitrite reduction to nitrogen. ACS Catal.2023, 13, 87–98.(3) Using PdCu electrocatalysts for nitrate conversion, the study synthesizes palladium nanocubes with varied copper coverage. Partial copper-coated cubes excel in NO_3_^–^ reduction, while full coverage favors NO_2_^–^ to NH_4_^+^ conversion. DFT calculations suggest NO* spillover from Cu to Pd enhances N_2_ selectivity.
- HuangP.-W.; HatzellM. C.Prospects and good experimental practices for photocatalytic ammonia synthesis. Nat. Commun.2022, 13, 7908.3656438210.1038/s41467-022-35489-7PMC9789054(4) Challenges arise from the absence of efficient photocatalysts and inconsistent data. This work identifies causes of these discrepancies, proposes solutions, and underscores key challenges in the field.

## Introduction

To meet the global net zero goals for 2050 related to carbon emissions, the industrial sector will need to achieve widespread electrification. Within the chemical sector, this will require significant advances in reactor engineering which allow for electrical heating. When new reactor architectures are not enough, new renewable-driven catalysts and separation-based technologies may be essential. Two emerging catalytic approaches that rely only on electricity and renewable energy are photocatalysis and electrocatalysis.^5,6^ Although photocatalysis has mainly remained in the conceptual phase,^7^ electrocatalysis is readily used today for the synthesis of a number of chemicals, including caustic soda, chlorine gas, and hydrogen. This progress in the fields of photocatalysis and electrocatalysis coupled with the growing global needs has sparked a rapid interest in exploring how photocatalysis can be used to synthesize more complex chemicals.^8^ Specifically, there has been a growing focus on the use of photocatalysis and electrocatalysis to aid in addressing the traditionally hard-to-decarbonize chemicals, which are often based on nitrogen (ammonia, hydrazine, and amines) and carbon (ethylene, syngas, and jet fuels).

Ammonia is of interest as 176 tons of ammonia are produced yearly, with 80% of this used to produce synthetic fertilizers (ammonia nitrate, urea, ammonium sulfate, etc.).^5,8^ Electrocatalytic and photocatalytic ammonia production has been heavily investigated over the last few decades. However, low yields, inconsistent performance, and high costs still plague the field. Despite these challenges, significant gains have been made in the production of ammonia using a lithium-mediated reaction. In this catalytic process, an electrochemical potential drives the formation of lithium nitride. As nitrogen activation is generally the most challenging step in the conversion of nitrogen to ammonia, the highly reactive nature of lithium aids in decreasing this energy barrier. In this approach, lithium nitride is hydrogenated using a range of proton sources.

Beyond ammonia, the direct formation of specialty nitrogen and carbon-containing chemicals is of growing interest. The ability to form C–N bonds for the production of fine chemicals using photochemical and electrochemical systems is worth pursuing, as it could allow for higher financial gains and allow for an easier entry into the market place. For instance, ammonia today is $400–$1000/ton, whereas sodium cyanide (used for mining) is about $1500/ton, and methylamine (used for carbon capture) is on the order of $3000/ton. Direct synthesis of urea from nitrogen and CO_2_ is also appealing, as this would promote process intensification. Urea today is synthesized using ammonia as a feedstock by the Bosch-Meiser process. This process requires high temperatures (180 °C) and pressure (∼150 bar).^9^ Therefore, direct use of nitrogen or waste nitrate, both abundant and more sustainable alternatives, would allow for ammonia to be formed as an intermediate during the co-reduction process. Incorporating these alternative reagents into our processes is not merely an academic endeavor; it is a tangible step toward a greener chemical industry. Using such an approach would eliminate the need for direct ammonia synthesis in a separate reactor and could help decrease the carbon emissions associated with the process. Thus, we outline the progress that has been made in photochemical and electrochemical synthesis technologies for urea production.

## Photochemical C–N Formation: Implications for Ammonia Synthesis

### Photochemical Reduction of Nitrogen and Carbonacous Species

Photochemical nitrogen transformations were initially pioneered by a prominent Indian soil scientist, N.R. Dhar, in the 1930s–40s.^10^ This early work focused on illuminating sterile soils with differing hydrocarbon concentrations. These early works, while far from conclusive, hinted that the rate of abiotic ammonia synthesis and oxidation (nitrification) increased in the presence of sunlight. In addition to nitrification, abiotic N_2_ photofixation has been shown to occur in natural environments.^11^ The role of carbonaceous species such as cellulose, plant residues, fats, and molasses was shown to be important in increasing the rate of nitrogen photofixation. These results remained unverified until 1977, when Schrauzer and Guth also began investigating photocatalyst ammonia synthesis in natural minerals.^7,12^ However, the specific role of carbon has not been elucidated and remains a significant knowledge gap.

Since these early investigations, there has been a growing interest in understanding nitrogen photofixation in the solar fuels community.^13−17^ While there have been some fundamental investigations aimed at understanding reaction mechanisms,^13,18,19^ the majority of published articles have focused on material selection and optimization. Furthermore, most studies have also focused on improving individual performance values such as quantum efficiency, yield, and rate of production.

Carbon-based catalysts, such as g-C_3_N_4_, are widely discussed as potential materials for photocatalytic nitrogen fixation due to their material stability, cost-effectiveness, and metal-free properties.^20−22^ Their nitrogen-containing characteristics may, however, introduce adventitious ammonia during the reaction, and such contamination could lead to false-positive results.^4,23,24^ More recently, studies suggest that carbon, in addition to serving as a substrate, appears to play a more direct role in the process of photocatalytic nitrogen fixation.^1,25^ Near-ambient-pressure XPS (AP-XPS) was used to probe photocatalyst surfaces in operando. A rutile (110) model surface was chosen based on prior observation that ammonia yields correlate with the amount of rutile,^26^ and experiments were carried out with in situ illumination by ultraviolet/visible light in the presence of 300 mTorr of N_2_ at 200 °C. The results for the as-prepared rutile (110) crystal indicate the emergence of a peak at 398 eV in the N 1s spectrum upon illumination.^18^ This value has previously been attributed to reduced nitrogen N_red_ (NH_3_ = 398.8 eV).^27,28^ This provides direct evidence of a photoinduced interaction between the titania surface and nitrogen. The results also indicate that adventitious carbon was present in the sample, a typical result for titania,^29,30^ while oxygen vacancies are not detected at the Ti 2p peak. When adventitious carbon was removed, the N1 peak could not be detected at illumination. These findings suggest that carbon may play a role in mediating an interaction between nitrogen and titania.

Computational models using density functional theory (DFT) have been applied to the problem of understanding the AP-XPS results. Specifically, significant exploration has been done with regard to the N_2_ binding energy on a range of oxygen/titanium defects and carbon additions/substitutions. The results illustrate a trade-off of reactivity and stability, where the most relevant sites exhibit maximum stability for a given N_2_ adsorption energy. There are several carbon-based active sites that exhibit N_2_ adsorption free energies of ∼2 eV, providing further theoretical support for the hypothesis that carbon facilitates interaction between TiO_2_ and N_2_. This is also consistent with previous reports of carbon radicals at the edges of graphene that strongly interact with nitrogen^31^ and carbon-based catalysts for photocatalytic nitrogen fixation.^8^

### Photochemical Coreduction of Nitrogenous Species and CO_2_

Photochemical synthesis of urea was first demonstrated by Yoneyama et al. in 1998.^32^ They utilized TiO_2_ nanocrystals (immobilized in polyvinylpyrrolidinone film) as the photocatalyst to coreduce CO_2_ and NO_3_^–^ (from LiNO_3_) to form urea in the propylene carbonate solution containing isopropanol as a hole scavenger. In addition to urea, the byproducts formed included methanol, ammonia, and a small amount of hydrogen. Acetone, generated from the oxidation of isopropanol, is the only observed oxidation product. The sum of the quantum efficiencies of the reduction products corresponds closely to the quantum efficiencies of the oxidation product (acetone), suggesting that these five reactions essentially cover the entire photocatalytic reaction. It should be noted that the reduction of NO_3_^–^ was hypothesized as the rate-determining step in the urea photosynthesis reaction, as evidenced by the significant increase in urea yield when the nitrogen source is switched to NO or NH_2_OH, both of which are intermediates in the reduction reaction of NO_3_^–^. Additionally, they observed that urea participated in oxidation reactions, leading to urea decomposition. They attributed the superior activity of the film catalyst compared to the colloidal catalyst to the better separation of the urea generated from TiO_2_ particles in the film catalyst.

In their follow-up study, the effect of solvents with different polarities,^33^ including ethylene glycol monoethyl ether, acetonitrile, sulfolane, and water, on the photochemical synthesis of urea was discussed. In this study similar reaction conditions were used; however, instead of methanol, formate, and CO were observed as byproducts of the reduction of CO_2_. From the results, the highest polarity solvent, which is water, yielded the highest amounts of urea. This could be attributed to the high polarity solvents favoring the dissociation of LiNO_3_, thereby allowing more NO_3_^–^ to participate in the reduction reaction. Aligned with their previous report, the primary conclusion of this study is that the reduction of NO_3_^–^ is the rate-determining step in the photocatalytic production of urea. Similar conclusions have been reported in other studies as well.^34,35^

Despite the inspiring findings, early studies lacked discussion of the reaction mechanism. It was not until 2011 that Srinivas first proposed a reaction pathway in his study on the photocatalytic synthesis of urea.^36^ In this study, zeolite-supported titania (or iron titanates) was used as a photocatalyst, and different hole scavengers (isopropanol and oxalic acid) were investigated for their effects on the yield and selectivity of urea. Additionally, carbonaceous hole scavengers in this work not only served as electron donors but also underwent further oxidation to produce CO_2_, thus serving as a carbon source. In their proposed mechanism, NO_3_^–^ is first reduced to NH_3_, which then reacts with CO_2_ to generate an unstable C–N linkage intermediate (NHCO), leading to the formation of formamide (HCONH_2_). Further transformations, including hydroxylation, amination, and dehydration reactions, occur to eventually yield urea. It should be noted that there is no experimental evidence supporting the presence of key intermediates such as NHCO and HCONH_2_. Therefore, the mechanism underlying the formation of urea during photochemical reactions is not yet clear.

**Figure 1:**
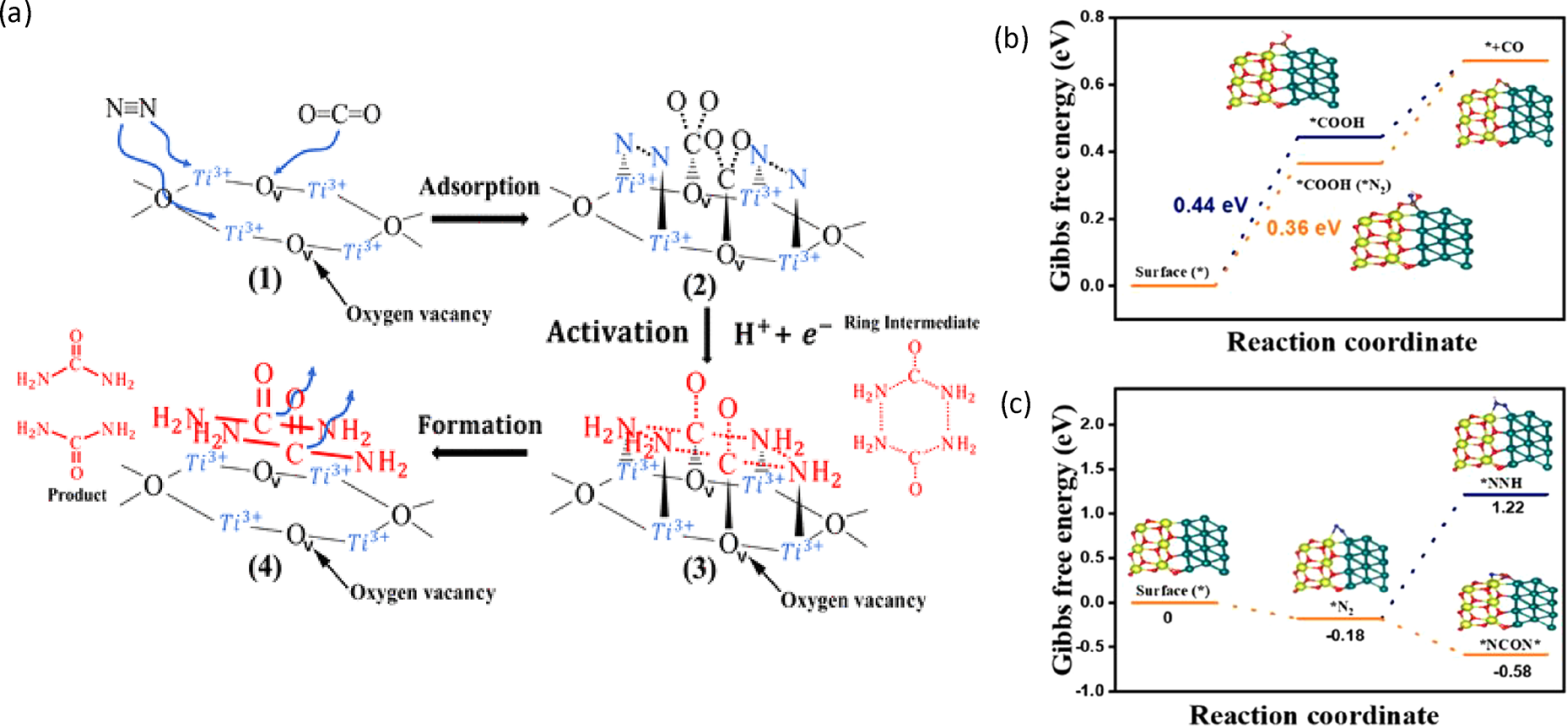
(a) Mechanism of photocatalytic coreduction of N_2_/CO_2_ to form CO(NH_2_)_2_. Reprinted from ref (37). Copyright 2021 American Chemical Society. (b) Free energy diagram of CO_2_ reduction with and without N_2_ adsorption on the photocatalyst. (c) Free energy diagram of N_2_ adsorption and further activation on the photocatalyst. Reprinted with permission from ref (39). Copyright 2023 Royal Society of Chemistry.

For unknown reasons, the photochemical synthesis of urea did not garner further attention in the following 10 years. It was not until 2021 when Maimaiti et al. utilized oxygen vacancy-rich TiO_2_ loaded on carbon nanotubes with Fe cores (Ti^3+^-TiO_2_/Fe-CNTs) as the catalyst and achieved the coreduction of N_2_ and CO_2_ into urea in water without the addition of a hole scavenger.^37^ The authors identified Ti^3+^ sites and the adjacent oxygen vacancy serves as the active center for N_2_ and CO_2_, respectively. Adsorbed N_2_ and CO_2_ are further activated by photogenerated electrons, forming six-membered cyclic intermediates (H_2_NCONH_2_)_2_, which eventually evolved into urea (Figure 1a). Similar dual-site activation mechanisms have also been reported in different material systems, such as CdS/BiOBr composites, where Cd^2+^ in CdS and oxygen vacancies in BiOBr adsorb and activate N_2_ and CO_2_, respectively, leading to the formation of crucial intermediate (*HNCONH*).^38^

Currently, no experimental evidence can directly support this mechanism, and there are unclear and contradictory results. For instance, when the environment is switched from CO_2_ + N_2_ to CO_2_ + NH_3_, BiOBr hardly produces urea, while CdS generates a good amount of urea, even more than CdS/BiOBr composites. These results contradict the viewpoint that CO_2_ is activated through the oxygen vacancies of BiOBr. The authors have proposed an alternative pathway alongside photocatalysis, suggesting that urea is also formed through a thermocatalytic reaction of reduced ammonia with CO_2_ on CdS. However, considering this reaction typically occurs under high temperature and pressure conditions (100–200 atm and 180 °C) in industry,^36^ whether the thermocatalytic pathway significantly contributes to urea synthesis in ambient photocatalytic reactions requires further investigation. Despite the presence of some obscure aspects, this mechanism serves as an important starting point for molecular-level understanding of the photocatalytic synthesis of urea. In the most recent study, Pd-decorated CeO_2_ was employed as a photocatalyst for the photoinduced coreduction of N_2_ and CO_2_ into urea.^39^ The DFT calculation revealed that the adsorbed N_2_ (*NN*, side-on adsorption structure) can promote the reduction of CO_2_ to CO, and the adsorbed N_2_ can effectively bind with the reduced CO to further form the key intermediates (i.e., *NCON* and *HNCONH*) and evolve into urea (Figure 1b,c). In this emerging field, the current crucial first step is to establish a reliable urea detection method, confirming that urea is generated from the catalytic pathway rather than due to measurement errors.^40^ In addition, more effort is needed to directly observe critical intermediates. For instance, in situ Fourier-transform infrared spectroscopy (FTIR) can be employed to probe the formation/evolution of C–N bonds on the catalyst surface under irradiation. These insights will contribute to establishing a more comprehensive mechanism for the photochemical synthesis of urea.

## Electrochemical C–N Formation: Implications for Urea Synthesis

Urea is synthesized through the Bosch-Meiser process, which primarily involves two reactions: (i) the formation of ammonium carbamate from ammonia and carbon dioxide (eq 1), followed by (ii) the decomposition of carbamate into urea and water (eq 2).^41^ The coupling of the C–N bond is a critical step in the formation of urea.
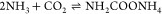
1
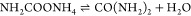
2

Electrochemical urea formation requires the use of electrocatalysts that can efficiently reduce carbon dioxide to carbon-based intermediates (eqs 3 and 4) and nitrogenous reactants (NOx and N_2_) to the ammonia intermediate (eqs 5, 6, and 7) concurrently, all while minimizing the hydrogen evolution reaction (HER). Despite numerous studies in the field, the precise reaction mechanisms governing these simultaneous reductions remain unclear. One proposed mechanism involves the coupling of C–N through *NH_2_ and *CO intermediates (eq 8).^42^ This is supported by the observation that the reduction of NH_3_ and CO, as opposed to CO_2_ and NO_3_^–^, does not result in urea production.^43^ The coreduction of NO_2_^–^ and CO_2_ to urea involves a 12-electron transfer (eq 9), while the coreduction of NO_3_^–^ and CO_2_ to urea involves an 16-electron transfer (eq 10).
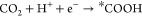
3
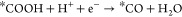
4
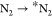
5
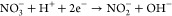
6
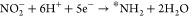
7
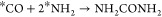
8
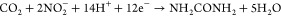
9
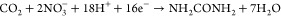
10

**Figure 2:**
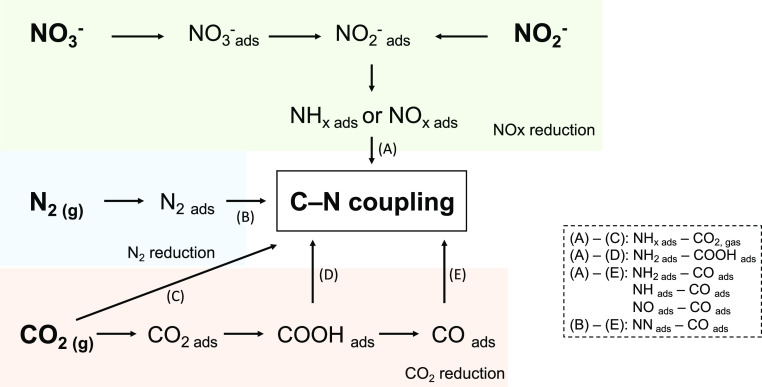
Possible intermediates and reaction pathways for C–N coupling in urea synthesis: (A–C),^45^ (A–D),^46^ (A–E),^42−45,47^ and (B–E).^48^

Molecular dynamics simulations revealed a favorable pathway for urea formation, which presents a lower kinetic barrier for C–N bond formation than the protonation of the *NH intermediate for ammonia formation. The pathway is as follows: *CO + 2*NH → *NH–CO + *NH → *CO(NH)_2_ → *NH_3_–CO–NH → (NH_2_)_2_CO. At more negative potentials (less than −0.75 V), competing reactions start to take place, which reduces the efficiency of urea production and favors the formation of other products.^44^ Besides these reaction pathways, multiple potential reaction intermediates and pathways may exist (Figure 2). While these mechanisms remain under debate, elucidating them is crucial for understanding urea production.

### Electrochemical Coreduction of NOx-CO_2_

Catalyst design such as metal alloys, doping, oxygen vacancies (OVs), and defects allows the tuning of electronic and geometric properties for the efficient NOx-CO_2_ coreduction.

Metal alloying, such as Au and Cu, facilitates the adsorption of the reactant by reducing the electron density of Au atoms.^42^ Additionally, in Cu–Zn core–shell structures, the induced electron transfer from Zn shell to Cu core can facilitate the formation of *CO and *NH_2_, promoting C–N coupling.^47^

Doping offers a beneficial approach for adjusting the atomic structure of both metal and nonmetal catalysts, controlling the binding energy of the intermediates. For example, Te doping on Pd nanocrystals shows stronger CO_2_ adsorption than pure Pd nanocrystals, this influence in the promotion of CO_2_RR. Te presence also influences the adsorption of CO and promotes *CO and *NH_2_ reaction to urea. This reduces carbon poisoning in the catalyst.^43^ OVs in CeO_2_ help stabilize the ·NOCO intermediate (formed through *NO and *CO coupling) detected by *in situ* sum frequency generation spectroscopy.^49^ OVs in OV-InOOH catalyst favor the In–O breakage, lowering the energy for *CO_2_NH_2_ protonation, which is the potential determining step (PDS) of NO_3_^–^ and CO_2_ coreduction to urea as shown by synchrotron radiation-Fourier transform infrared spectroscopy (SR-FTIR).^50^ OVs in TiO_2_(101) form Ti_3_^+^ sites that bind N atoms favoring its reduction. The Cu sites both adsorb and reduce CO_2_,^51^ while also resulting in oxygen vacancies due to the substitution of a species with lower charge (Cu for Ti).^52^

Furthermore, certain defects and design strategies can complementarily synthesize a catalyst harboring active sites specific to multiple intermediates or reaction steps. For instance, a diatomic Fe–Ni catalyst has demonstrated superior activity toward urea synthesis compared to its Fe-SAC and Ni-SAC counterparts due to the provision of an active, activation, and coupling site conducive to C–N coupling.^53^

### Electrochemical Coreduction of N_2_ and CO_2_

The direct coreduction of gaseous CO_2_ and N_2_ into urea offers considerable benefits due to their abundant presence on Earth. However, this approach also brings challenges.The yield rate of urea from a N_2_ reactant is potentially lower compared to NOx, largely due to strong triple bond of N_2_. Additionally, there are obstacles, such as weak molecule adsorption, slow bond cleavage, and the complex C–N coupling reaction in electrocatalysis. To overcome these hurdles, extensive research is being conducted to optimize operational parameters and engineer efficient catalysts that enhance the production of urea from gaseous CO_2_ and N_2_. In particular, a study conducted with a high pressure cell (60 bar) system using polyaniline (PAni) and polypyrrole (PPy) coated platinum electrodes demonstrated a significant advancement.^54^ Researchers achieved selective urea production with a Faradaic efficiency (FE) of 7.1% under this high pressure condition (30 bar N_2_ + 30 bar CO_2_). Interestingly, the authors suggested that the production of urea follows the industrial urea process, which is operating at high pressure and temperature, involving the decomposition of ammonium carbamate (NH_2_COONH_4_ → NH_2_CONH_2_ + H_2_O), as supported by the analysis of the Tafel slope and activation energy.

However, under ambient conditions, the synthesis of urea involves more intricate pathways such as the adsorption and coupling of intermediates. Research addressing this complexity has demonstrated selective urea production (FE: 8.92%) by developing CuPd alloy nanoparticles in TiO_2_ nanosheets (Pd_1_Cu_1_/TiO_2_-400).^55^ Pd_1_Cu_1_/TiO_2_-400 exhibited a synergistic effect through the combination of bimetallic catalysts and optimized electronic structures, enhancing chemisorption and catalytic activity. Isotope-labeled operando SR-FTIR and DFT calculations supported the hypothesis that the C–N coupling process occurs through a thermodynamically spontaneous reaction between *N=N* and *CO.

Furthermore, significant strides in the design and engineering of catalysts have led to remarkable progress in the electrocatalytic conversion of N_2_ and CO_2_ into urea. These developments by the Guangjin Zhang group have led to a successful fine-tuning of the electronic properties in a variety of materials, including nickel borate,^56^ Bi–BiVO_4_ heterostructures,^48^ conductive metal–organic frameworks (MOFs),^57^ and perovskite heterostructured BiFeO_3_/BiVO_4_ hybrids.^58^ Specifically, they achieved a urea FE of 20.36% with a unique nickel borate catalyst, 12.55% with novel Mott–Schottky Bi-BiVO_4_ heterostructures, 17.18% with perovskite heterostructured BiFeO_3_/BiVO_4_ hybrids, and 48.97% with a novel conductive MOF Co-PMDA-2-mblM. These catalysts were designed to create local electrophilic and nucleophilic regions, facilitating targeted adsorption, activation, and coupling of N_2_ and CO_2_ molecules. This approach overcomes the major challenges associated with the poor chemisorption and coupling abilities of the reactant molecules.

Another strategy involves creating multiple active sites for the simultaneous reduction of both N_2_ and CO_2_ into urea under a similar potential range. In this regard, copper phthalocyanine nanotube (CuPc NTs), which have multiple active sites for both CO_2_RR and NRR, demonstrated selective urea production with a FE of 12.99%.^59^ Computational insights from DFT calculations suggested that the pyridinic-N1 and metal center in CuPc facilitate C–N bonding for urea through the coreduction of N_2_ to *NH and CO_2_ to *CO, respectively.

## Challenges and Future Prospects

**Figure 3:**
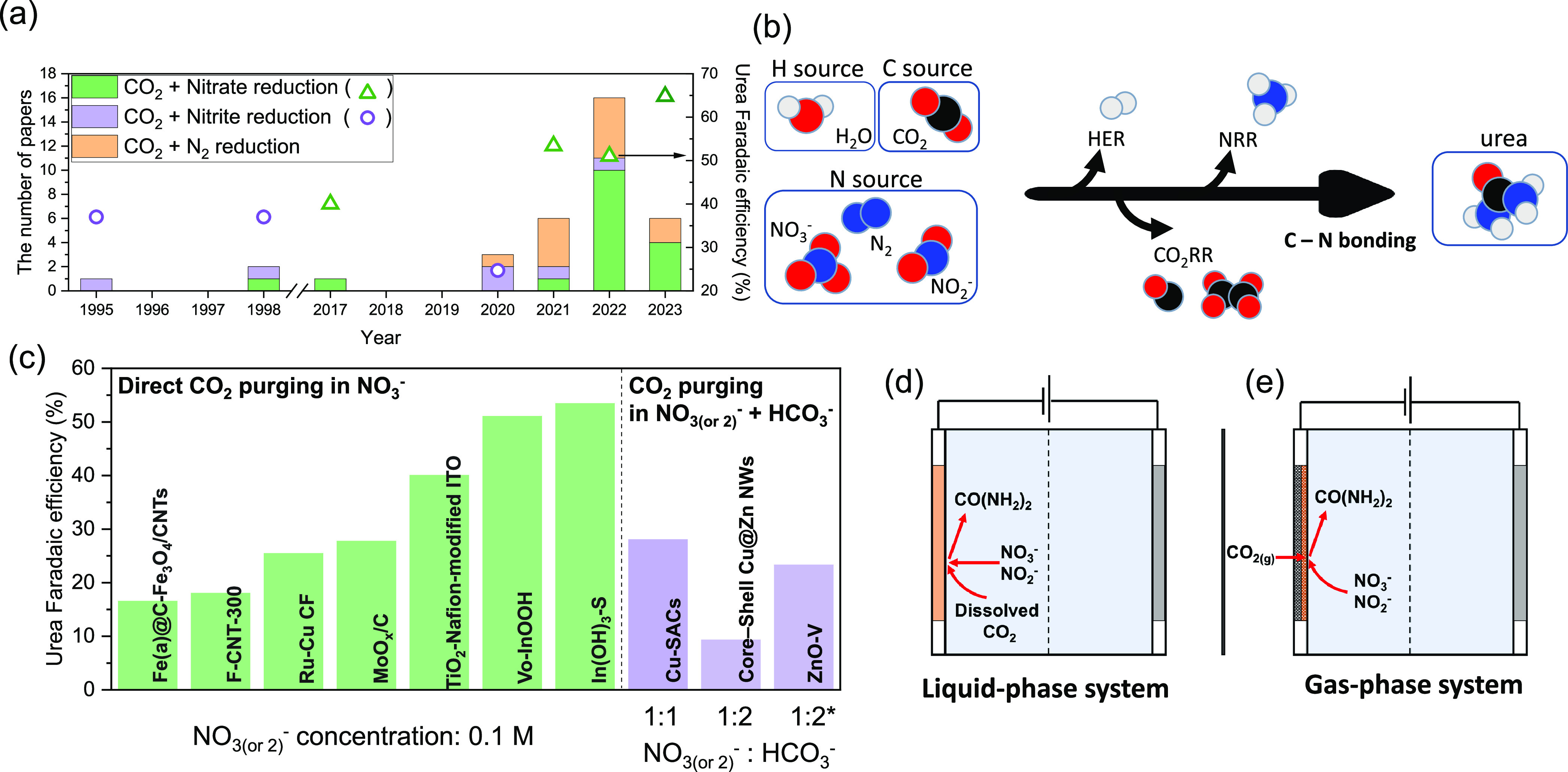
(a) Number of published papers on urea synthesis through coreduction of CO_2_ and NOx or N_2_ from 1995 to April 2023. The highest Faradaic efficiency achieved each year is represented on the right *y*-axis, with different shapes (△ and ○). (b) Schematic diagram of urea synthesis from hydrogen, carbon, and nitrogen sources. (c) Urea Faradaic efficiencies as a function of electrolytes, comparing direct CO_2_ purging in NO_3_^–^ and CO_2_ purging in HCO_3_^–^ with NO_3_^–^ (or NO_2_^–^) at a concentration of 0.1 M. * represents NO_2_^–^.^46,47,50,62−67^ Comparison between (d) liquid-phase and (e) gas-phase electrolysis systems for urea synthesis.

Since 2020, there has been a steady stream of research papers published and considerable progress made in improving the selectivity of urea production (Figure 3a). Despite these significant advancements, the selective and efficient synthesis of urea through photo/electrochemical coreduction of CO_2_ and nitrogenous reactants (NOx and N_2_) remains a challenging endeavor. Particularly, the generation of numerous intermediates and products, including carbon-, nitrogen-, and carbon-nitrogen coupled-compounds, and HER, hinders the selective and efficient production of urea (Figures 2 and 3b). This account has delved into a multitude of studies focusing on enhancing urea production performance. Drawing on these insights, we outline key considerations and directions for future research, including the (1) design of catalysts, (2) impact of electrolytes, and (3) choice of reactors. (1) Design of catalysts: Proper catalysts for urea synthesis should be capable of simultaneously adsorbing and reducing CO_2_ and nitrogenous species. These catalysts must possess adaptable electronic and geometric structures. The combined effects of these electronic and geometric properties, which include features such as nanostructures, mesostructures, defects, alloys, oxides, and vacancies, are crucial for determining the efficiency of urea production. The electrocatalytic performance for the direct synthesis of urea, through the coreduction of CO_2_ with NO_3_^–^ and CO_2_ with N_2_, is presented in Tables S1 and S2. To further optimize these catalyst designs, in-depth theoretical research employing tools like DFT calculations and microkinetic modeling is imperative. (2) The impact of electrolytes: The role of electrolytes in urea synthesis is often overlooked in numerous studies, yet they significantly influence activity. In recent comprehensive research into NOx and CO_2_ coreduction, two primary strategies stand out. In one method, CO_2_ is directly purged into the NOx solution, and in the alternative approach, CO_2_ is introduced into a buffer solution like HCO_3_^–^ (Figure 3c). These approaches result in varying electrolytic conditions, especially differences in pH, which in turn affect urea synthesis activity. Given that the urea production process demands 14 protons from NO_2_^–^ and 18 from NO_3_^–^ (eqs 9 and 10), a lower pH without buffer might appear promising. Yet, it is essential to consider that a low pH environment might not be ideal due to the risk of increased HER activity and decomposition of urea.^60^ Hence, deeper investigations into electrolytes, both buffered and nonbuffered, are necessary to fully understand their impact. (3) The choice of reactors: Selecting the appropriate reactor is essential for both managing mass transport limitations and optimizing electrolysis environments. The efficiency of urea production can vary significantly depending on whether one uses liquid-phase systems, such as H-type cells, or gas-phase systems like gas-diffusion electrode (GDE) systems (Figure 3d,e). Each reactor type offers unique electrolysis environments that influence solubility, reactant transport, and diffusion rates. Given the challenges posed by the low solubility of reactants, GDE systems tend to be more suitable for high performance urea production. Recent advancements have underscored the capability of GDE systems to notably enhance the urea reaction rate, reaching up to 115.25 mA/cm^2^).^61^ Besides GDEs, reactors like high-pressure and membrane electrode assembly (MEA) type cells also merit consideration. Overall, thorough evaluation and optimization of these reactor types are pivotal for advancing urea synthesis efficiency.

**Figure 4:**
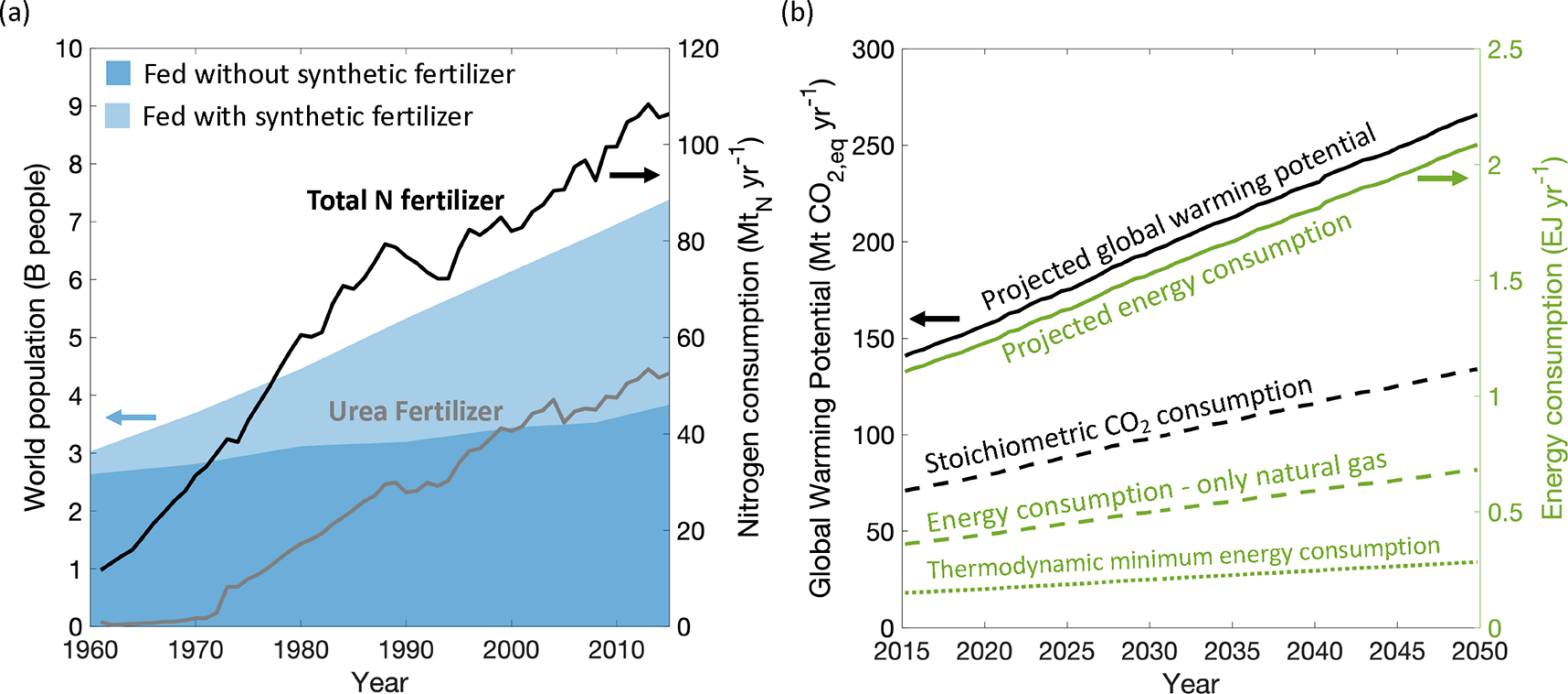
(a) World population increase between 1960 and 2015.^69^ Shaded regions indicate population fraction fed with and without synthetic fertilizer. (left axis) Total N fertilizer and urea fertilizer consumption worldwide between 1961 and 2015. (right axis) Data for total N fertilizer and urea (1973–2015) from IFA; data for urea (1961–1972) from FAO.^74,75^ (b) Projected global warming potential through 2050 based on moderate projection of population and fertilizer consumption growth, in comparison with theoretical consumption of CO_2_ if urea production used captured CO_2_. (left axis) Projected energy consumption through 2050 for global urea production. (right axis) Baseline calculations assumed an energy split of 70% natural gas to 30% coal for urea production.^78−87^ Green dashed line indicates 100% natural gas assumption. Green dotted line indicates thermodynamic minimum energy required.^88^

Continued improvement of these technologies will increasingly become crucial as synthetic fertilizers like urea are integral to sustaining current population growth (Figure 4a).^68−71^ By 2050, the global population is projected to reach 9.7 billion people, which combined with changing socio-economic trends, will increase the food demand by at least 54%.^72,73^ An increased dependence on urea is expected to maintain high crop yields in 2015, 50% of the applied fertilizer worldwide was urea, and it was estimated that 48% of the world’s population was fed using synthetic fertilizers.^69,74,75^ In addition, urea is increasingly being used in other sectors, such as animal feed, chemical synthesis, diesel exhaust fluid, thereby adding to the demand.^76,77^

In prior work, ammonia production was projected until 2050 using population growth and future fertilizer use assumptions.^78^ The moderate case, where the birth rates remain high and the nitrogen fertilizer per capita increases, can be used to calculate the urea projection as 55% of all ammonia goes to the production of urea (Figure 4b).^79^ The theoretical carbon footprint for urea can be calculated from stoichiometry (0.735–0.75 *t*_CO2_/*t*_urea_).^80,81^ However, even when carbon capture is implemented, the actual reported carbon footprint for urea production is non-negative, reaching 2.27 *t*_CO2,eq_/*t*_CO2,used_ for urea production plants using coal and 1.38 *t*_CO2,eq_/*t*_urea_ for those using natural gas.^82,83^ This discrepancy is due to energy-intensive processes that are required to form urea as well as losses in the system. By 2050, the global warming potential for urea production could reach 286 Mt_CO2,eq_/yr, which highlights the need to target the energy sources used throughout the process. For example, China produces 30% of the world’s urea using coal, resulting in a much larger carbon output than a comparable urea production using natural gas.^84−86^ We see a 67% decrease in energy consumption just by assuming that global urea production was made using natural gas.^87^ Additional improvements can be made by changing the CO_2_ source to renewable sources like biomass or direct air capture, thus achieving “green urea” and tending toward the thermodynamic minimum.^80^ Electrochemical processes currently cannot compete with the state-of-the-art energy requirements due to high total applied potentials and low urea output (additional information is included in the Supporting Information).

Achieving selective and efficient urea synthesis remains a significant challenge, mandating continued exploration of innovative approaches in catalyst development and operation optimization. This review is just the starting point, and there is much more to uncover in the realm of photochemical and electrochemical urea synthesis. Advancements in understanding the intricate relationship between reaction mechanisms, catalyst design, electrolyte choice, and reactors will open new research avenues. Future studies will bring us closer to resolving challenges in urea synthesis and enhance our understanding of CO_2_ utilization and nitrogen cycle management.

## Supporting Information Available

Future outlook: projections and assumptions; electrochemical co-reduction of CO_2_ and NO_3_^–^ (Table S1); electrochemical co-reduction of CO_2_ and N_2_ (Table S2); number of published papers on photosynthesis urea through co-reduction of CO_2_ and NO_3_^–^ or N_2_ from 1995 to April 2023 (Figure S1); and band diagram of photocatalysts used for photocatalytic urea production (Figure S2) (PDF)
