# Supplementary material for: Progress in Photochemical and Electrochemical C–N Bond Formation for Urea Synthesis
Source: Acc Chem Res. 2023 Oct 19;56(21):2944–53. doi: 10.1021/acs.accounts.3c00424 (PMC10634294; doi:10.1021/acs.accounts.3c00424)
Supplement: Supplementary file 1 — ar3c00424_si_001.pdf [file ar3c00424_si_001.pdf]

Supporting information

Progress in Photochemical and Electrochemical C-N Bond Formation for Urea Synthesis

Hakhyeon Song,[†] *Danae A. Chipoco Haro,*[‡] *Po-Wei Huang,*[¶] *Luisa Barrera,*[†]
and Marta C. Hatzell[*†,¶]

†George W. Woodruff School of Mechanical Engineering, Georgia Institute of Technology,
Atlanta, Georgia, 30332, United States
‡School of Materials Science and Engineering, Georgia Institute of Technology, Atlanta,
Georgia, 30332, United States
¶School of Chemical and Biomolecular Engineering, Georgia Institute of Technology,
Atlanta, Georgia, 30332, United States
E-mail: marta.hatzell@me.gatech.edu
Phone: +1 404-385-45036

Future outlook: projections and assumptions

Using prior work, the projected market volume for ammonia was used to project the future market volume of urea: for a conservative estimate, we used the moderate case, where the birth rates remain high and the nitrogen fertilizer per capita increases;[1] the International Energy Agency estimates that 55% of all ammonia produced is consumed by urea production.[2]

The theoretical carbon footprint can be calculated from stoichiometry, reaching 0.735-0.75 Mt_{CO2}/Mt_{urea} (averaged for calculations), or 1.5919 $Mt_{CO2}/Mt_{urea\text{-}N}$ after molar mass fraction conversion.[3,4] However, larger carbon footprints for urea production (i.e. non-negative) have been reported and directly depend on the source of energy being used, with 70% of urea is produced using natural gas and 30% is produced using coal. This energy split was assumed from the following: China represents 30% of the world's urea production, and 94.4% of China's production uses coal (this has been rounded to 100%).[5–7] It has been reported that urea production releases of 2.27 $Mt_{CO2,eq}/Mt_{CO2,used}$ when coal is the source of energy[8] (equivalent to 3.61 $Mt_{CO2,eq}/Mt_{urea\text{-}N}$) and 1.382 $Mt_{CO2,eq}/Mt_{urea}$ when natural gas is used[9] (2.96 $Mt_{CO2,eq}/Mt_{urea\text{-}N}$), even with carbon capture being implemented. Following these ratios, we can calculate the actual global warming potential from urea production (Eq. (S1)):

$$\mathrm{GWP}\left[\frac{\mathrm{Mt_{CO2,eq}}}{\mathrm{yr}}\right] = \mathrm{Urea\ production}\left[\frac{\mathrm{Mt_{urea-N}}}{\mathrm{yr}}\right] * (0.3 * 3.61 + 0.7 * 2.96) \quad \text{(S1)}$$

Similarly, we can estimate the energy consumed from the projected urea production, where there is again a significant dependence on the source of energy used. The energy reported for urea production using coal is 29.712 GJ/t_{urea} when including the urea synthesis and waste treatment, not the transport or extraction of raw materials.[5] For natural gas, the energy for the urea forming process reaches 8.10 GJ/t_{urea}.[10] The thermodynamic minimum energy was calculated using the activation energy required for the urea synthesis process, 94.5 kJ/mol, and the total molar amount of urea projected to be made.[11]

Comparing these projected values to photoelectrochemical and electrochemical processes is non-trivial. As the photoelectrochemical processes harness solar energy to drive the synthesis reaction, the additional energy requirements would be coming from the other elements of the system, such as pumping. These estimates are outside the scope of this work but would be an interesting avenue to pursue to test the economic viability of a photoelectrochemical process. For the electrochemical processes, the energy requirements of the synthesis processes can be estimated using the reported current density, total cell potential, surface area of the catalyst (SA), duration of experiment, and mass of formed urea:

$$\mathrm{Energy}\left[\frac{\mathrm{EJ}}{\mathrm{yr}}\right] = \mathrm{Urea\ production}\left[\frac{\mathrm{Mt_{urea-N}}}{\mathrm{yr}}\right] * \frac{j\left[\frac{\mathrm{A}}{\mathrm{m^2}}\right] V_{\mathrm{tot}}[\mathrm{V}] * \mathrm{SA}[\mathrm{m^2}] * t[\mathrm{s}]}{m_{urea}[\mathrm{Mt_{urea-N}}]} \quad \text{(S2)}$$

Huang et al is one of the few papers that actually report the total cell voltage, with $j = 40\ mA/cm^2$, $V_{tot} = 2.7$ V, and the assumed geometric surface area SA = 1 cm^2 and t = 1 hour = 3600 s.[12] The mass of urea m_{urea} was estimated using the reported yield rate (15.13 mmol/g/hr), mass catalyst (1.1 mg), as well as the molar mass (28 g_N/mol_{urea}). Using Eq. (S2), we find energy requirements 35-times larger than the projected energy consumption as shown in Figure 4. This highlights the need to improve the urea synthesis process for both output and energy consumption.

Table S1. Electrochemical co-reduction of CO_2 and NO_3^-.

Catalyst	Reactants		Electrolyte	Reactor	Faradaic efficiency (%)	Reference
	carbon	nitrogen				
**TiO_2/Nafion**	CO_2 gas	0.1 M KNO_3	0.1 M KNO_3	gas-tight H-type cell	40	13
**Ru-Cu CF**	CO_2 gas	0.1 M KNO_3	0.1 M KNO_3	batch cell (undivided cell)	25.4	14
**AuPd**	CO_2 gas	0.025 M KNO_3	0.075 M KHCO3	gas-tight H-type cell	15.6	15
**Oxide-Derived Core–Shell Cu@Zn Nanowires**	CO_2 gas	0.1 M KNO_3	0.2 M $KHCO_3$	H-type cell	9.28	16
**Fe-Ni/N-doped carbon**	CO_2 gas	0.05 M KNO_3	0.1 M $KHCO_3$	H-type cell	17.8	17
**Fe(a)@CFe_2O_4/ CNT**	CO_2 gas	0.1 M KNO_3	0.1 M KNO_3	2-chamber cell	16.5	18
**F-CNT**	CO_2 gas	0.1 M KNO_3	0.1 M KNO_3	H-type cell	18	19
**Cu**	CO_2 gas	0.05 M KNO_3	0.1 M $KHCO_3$	H-type cell	3	20
**Cu SAC**	CO_2 gas	0.1 M KNO_3	0.1 M $KHCO_3$	H-type cell	28	21
**Cu**	CO_2 gas	0.02 M KNO_3	0.2 M $KHCO_3$	gas-diffusion electrode	22	22
**Zn**	CO_2 gas	0.02 M KNO_3	0.2 M $KHCO_3$	gas-diffusion electrode	35	22
**Cu with atomic-scale spacings (ds)**	CO_2 gas	0.1 M KNO_3	1 M KOH	gas-diffusion electrode	51.9	23
**MoO_x/C**	CO_2 gas	0.1 M KNO_3	0.1 M KNO_3	H-type cell	27.7	24
**InOOH-OV**	CO_2 gas	0.1 M KNO_3	0.1 M KNO_3	H-type cell	51	25

Table S2. Electrochemical co-reduction of CO_2 and N_2.

Catalyst	Reactants (CO_2:N_2)		Electrolyte	Reactor	Faradaic efficiency (%)	Reference
	Pressure	ratio				
**Polyaniline (PAni) and polypyrrole (PPy) coated platinum**	60 bar (30 bar + 30 bar)	1:1	0.1 M Li_2SO_4/0.03M H^+	Batch type High pressure cell	7.1	26
**PdCu alloy nanoparticles on TiO_2**	ambient	1:1	0.1 M $KHCO_3$	H-type cell	8.92	27
**$Ni_3(BO_3)_2$**	ambient	1:1	0.1 M $KHCO_3$	H-type cell	20.36	28
**novel Mott–Schottky Bi-$BiVO_4$ heterostructures**	ambient	1:1	0.1 M $KHCO_3$	H-type cell	12.55	29
**MOF Co–PMDA–2-mbIM**	ambient	1:1	0.1 M $KHCO_3$	H-type cell	48.97	30
**$BiFeO_3$/$BiVO_4$**	ambient	1:1	0.1 M $KHCO_3$	H-type cell	17.18	31
**copper phthalocyanine nanotubes (CuPc NTs)**	ambient	1:1	0.1 M $KHCO_3$	H-type cell	12.99	32

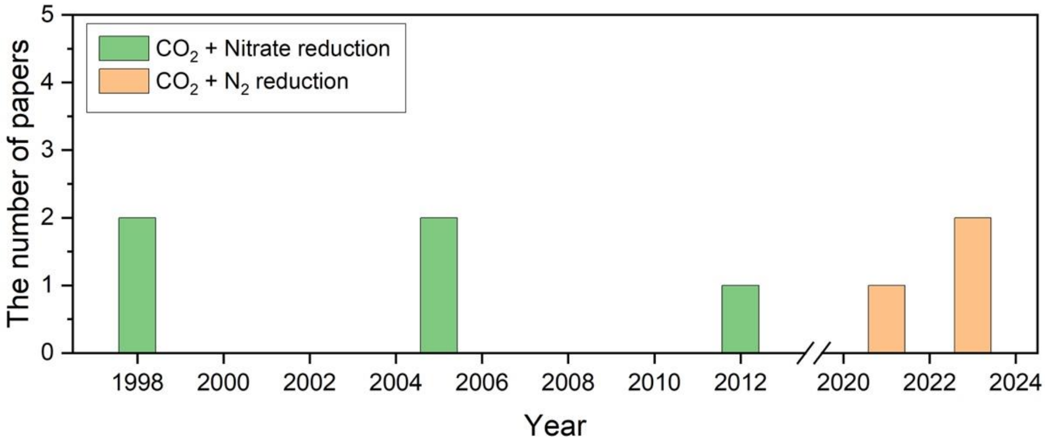

Figure S1: The number of published papers on photosynthesis urea through co-reduction of CO_2 and NO_3^- or N_2 from 1995 to April 2023.

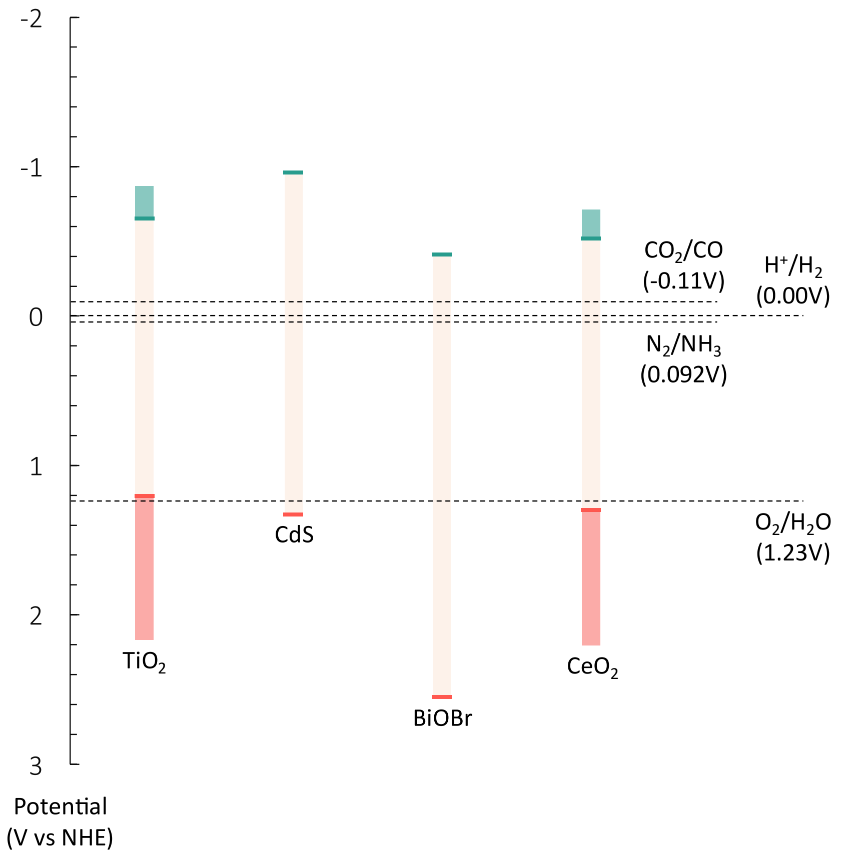

Figure S2: Band diagram of photocatalysts used for photocatalytic urea production. The width of conduction/valence bands shows range of values were reported. Band edges of defective TiO_2, CdS and BiOBr are taken from other studies.[33–35]

References

(1) Lim, J.; Fernández, C. A.; Lee, S. W.; Hatzell, M. C. Ammonia and Nitric Acid Demands for Fertilizer Use in 2050. *ACS Energy Lett.* **2021**, *6* (10), 3676–3685. https://doi.org/10.1021/acsenergylett.1c01614.

(2) International Energy Agency. *Ammonia Technology Roadmap: Towards More Sustainable Nitrogen Fertiliser Production*; OECD, 2021. https://www.oecd-ilibrary.org/energy/ammonia-technology-roadmap_f6daa4a0-en (accessed 2023-07-17).

(3) Milani, D.; Kiani, A.; Haque, N.; Giddey, S.; Feron, P. Green Pathways for Urea Synthesis: A Review from Australia's Perspective. *Sustain. Chem. Clim. Action* **2022**, *1*, 100008. https://doi.org/10.1016/j.scca.2022.100008.

(4) Muradov, N. Existing Industrial CO_2 Utilization Processes. In *Liberating Energy from Carbon: Introduction to Decarbonization*; Lecture Notes in Energy; Springer New York: New York, NY, 2014; Vol. 22, pp 330–332.

(5) Shi, L.; Liu, L.; Yang, B.; Sheng, G.; Xu, T. Evaluation of Industrial Urea Energy Consumption (EC) Based on Life Cycle Assessment (LCA). *Sustainability* **2020**, *12* (9), 3793. https://doi.org/10.3390/su12093793.

(6) S&P Global Commodity Insights. *Urea Fertilizer Market and Price Analysis*. S&P Global. https://www.spglobal.com/commodityinsights/en/ci/products/fertilizers-urea.html (accessed 2023-07-13).

(7) IEA. *How the energy crisis is exacerbating the food crisis*. IEA. https://www.iea.org/commentaries/how-the-energy-crisis-is-exacerbating-the-food-crisis (accessed 2023-07-13).

(8) Parsons Brinckerhoff. *Accelerating the Uptake of CCS: Industrial Use of Captured Carbon Dioxide*; Global CCS Institute, 2011.

(9) Shirmohammadi, R.; Aslani, A.; Batuecas, E.; Ghasempour, R.; Romeo, L. M.; Petrakopoulou, F. A Comparative Life Cycle Assessment for Solar Integration in CO_2 Capture Utilized in a Downstream Urea Synthesis Plant. *J. CO_2 Util.* **2023**, *74*, 102534. https://doi.org/10.1016/j.jcou.2023.102534.

(10) Lako, P. *Energy Conservation Potential of the Nitrogen Fertiliser Industry*; ECN-E--09-011; Energy Research Centre of the Netherlands, 2009.

(11) Zhang, X.; Zhang, S.; Yao, P.; Yuan, Y. Modeling and Simulation of High-Pressure Urea Synthesis Loop. *Comput. Chem. Eng.* **2005**, *29* (5), 983–992. https://doi.org/10.1016/j.compchemeng.2004.10.004.

(12) Huang, Y.; Yang, R.; Wang, C.; Meng, N.; Shi, Y.; Yu, Y.; Zhang, B. Direct Electrosynthesis of Urea from Carbon Dioxide and Nitric Oxide. *ACS Energy Lett.* **2022**, *7* (1), 284–291. https://doi.org/10.1021/acsenergylett.1c02471.

(13) Saravanakumar, D.; Song, J.; Lee, S.; Hur, N. H.; Shin, W. Electrocatalytic Conversion of Carbon Dioxide and Nitrate Ions to Urea by a Titania–Nafion Composite Electrode. *ChemSusChem* **2017**, *10* (20), 3999–4003. https://doi.org/10.1002/cssc.201701448.

(14) Qin, J.; Liu, N.; Chen, L.; Wu, K.; Zhao, Q.; Liu, B.; Ye, Z. Selective Electrochemical Urea Synthesis from Nitrate and CO_2 Using In Situ Ru Anchoring onto a Three-Dimensional Copper Electrode. *ACS Sustainable Chem. Eng.* **2022**, *10* (48), 15869–15875. https://doi.org/10.1021/acssuschemeng.2c05110.

(15) Wang, H.; Jiang, Y.; Li, S.; Gou, F.; Liu, X.; Jiang, Y.; Luo, W.; Shen, W.; He, R.; Li, M. Realizing Efficient C-N Coupling via Electrochemical Co-Reduction of CO_2

and NO3- on AuPd Nanoalloy to Form Urea: Key C-N Coupling Intermediates. *Appl. Catal. B-Environ.* **2022**, *318*, 121819. https://doi.org/10.1016/j.apcatb.2022.121819.
(16) Meng, N.; Ma, X.; Wang, C.; Wang, Y.; Yang, R.; Shao, J.; Huang, Y.; Xu, Y.; Zhang, B.; Yu, Y. Oxide-Derived Core–Shell Cu@Zn Nanowires for Urea Electrosynthesis from Carbon Dioxide and Nitrate in Water. *ACS Nano* **2022**, *16* (6), 9095–9104. https://doi.org/10.1021/acsnano.2c01177.
(17) Zhang, X.; Zhu, X.; Bo, S.; Chen, C.; Qiu, M.; Wei, X.; He, N.; Xie, C.; Chen, W.; Zheng, J.; Chen, P.; Jiang, S. P.; Li, Y.; Liu, Q.; Wang, S. Identifying and Tailoring C–N Coupling Site for Efficient Urea Synthesis over Diatomic Fe–Ni Catalyst. *Nat. Commun.* **2022**, *13* (1), 5337. https://doi.org/10.1038/s41467-022-33066-6.
(18) Geng, J.; Ji, S.; Jin, M.; Zhang, C.; Xu, M.; Wang, G.; Liang, C.; Zhang, H. Ambient Electrosynthesis of Urea with Nitrate and Carbon Dioxide over Iron-Based Dual-Sites. *Angew. Chem. Int. Ed.* **2023**, *62* (6), e202210958. https://doi.org/10.1002/anie.202210958.
(19) Liu, X.; Kumar, P. V.; Chen, Q.; Zhao, L.; Ye, F.; Ma, X.; Liu, D.; Chen, X.; Dai, L.; Hu, C. Carbon Nanotubes with Fluorine-Rich Surface as Metal-Free Electrocatalyst for Effective Synthesis of Urea from Nitrate and CO_2. *Appl. Catal. B-Environ.* **2022**, *316*, 121618. https://doi.org/10.1016/j.apcatb.2022.121618.
(20) Krzywda, P. M.; Paradelo Rodríguez, A.; Benes, N. E.; Mei, B. T.; Mul, G. Carbon-Nitrogen Bond Formation on Cu Electrodes during CO_2 Reduction in NO_3^- Solution. *Appl. Catal. B-Environ.* **2022**, *316*, 121512. https://doi.org/10.1016/j.apcatb.2022.121512.
(21) Leverett, J.; Tran-Phu, T.; Yuwono, J. A.; Kumar, P.; Kim, C.; Zhai, Q.; Han, C.; Qu, J.; Cairney, J.; Simonov, A. N.; Hocking, R. K.; Dai, L.; Daiyan, R.; Amal, R. Tuning the Coordination Structure of Cu-N-C Single Atom Catalysts for Simultaneous Electrochemical Reduction of CO_2 and NO_3^- to Urea. *Adv. Energy Mater.* **2022**, *12* (32), 2201500. https://doi.org/10.1002/aenm.202201500.
(22) Shibata, M.; Yoshida, K.; Furuya, N. Electrochemical Synthesis of Urea on Reduction of Carbon Dioxide with Nitrate and Nitrite Ions Using Cu-Loaded Gas-Diffusion Electrode. *J. Electroanal. Chem.* **1995**, *387* (1), 143–145. https://doi.org/10.1016/0022-0728(95)03992-P.
(23) Shin, S.; Sultan, S.; Chen, Z.-X.; Lee, H.; Choi, H.; Wi, T.-U.; Park, C.; Kim, T.; Lee, C.; Jeong, J.; Shin, H.; Kim, T.-H.; Ju, H.; Chul Yoon, H.; Song, H.-K.; Lee, H.-W.; Cheng, M.-J.; Kwon, Y. Copper with an Atomic-Scale Spacing for Efficient Electrocatalytic Co-Reduction of Carbon Dioxide and Nitrate to Urea. *Energy Environ. Sci.* **2023**, *16* (5), 2003–2013. https://doi.org/10.1039/D3EE00008G.
(24) Lv, C.; Lee, C.; Zhong, L.; Liu, H.; Liu, J.; Yang, L.; Yan, C.; Yu, W.; Hng, H. H.; Qi, Z.; Song, L.; Li, S.; Loh, K. P.; Yan, Q.; Yu, G. A Defect Engineered Electrocatalyst That Promotes High-Efficiency Urea Synthesis under Ambient Conditions. *ACS Nano* **2022**, *16* (5), 8213–8222. https://doi.org/10.1021/acsnano.2c01956.
(25) Lv, C.; Zhong, L.; Liu, H.; Fang, Z.; Yan, C.; Chen, M.; Kong, Y.; Lee, C.; Liu, D.; Li, S.; Liu, J.; Song, L.; Chen, G.; Yan, Q.; Yu, G. Selective Electrocatalytic Synthesis of Urea with Nitrate and Carbon Dioxide. *Nat. Sustain.* **2021**, *4* (10), 868–876. https://doi.org/10.1038/s41893-021-00741-3.
(26) Kayan, D. B.; Köleli, F. Simultaneous Electrocatalytic Reduction of Dinitrogen and Carbon Dioxide on Conducting Polymer Electrodes. *Appl. Catal. B-Environ.* **2016**, *181*, 88–93. https://doi.org/10.1016/j.apcatb.2015.07.045.

(27) Chen, C.; Zhu, X.; Wen, X.; Zhou, Y.; Zhou, L.; Li, H.; Tao, L.; Li, Q.; Du, S.; Liu, T.; Yan, D.; Xie, C.; Zou, Y.; Wang, Y.; Chen, R.; Huo, J.; Li, Y.; Cheng, J.; Su, H.; Zhao, X.; Cheng, W.; Liu, Q.; Lin, H.; Luo, J.; Chen, J.; Dong, M.; Cheng, K.; Li, C.; Wang, S. Coupling N_2 and CO_2 in H_2O to Synthesize Urea under Ambient Conditions. *Nat. Chem.* **2020**, *12* (8), 717–724. https://doi.org/10.1038/s41557-020-0481-9.

(28) Yuan, M.; Chen, J.; Xu, Y.; Liu, R.; Zhao, T.; Zhang, J.; Ren, Z.; Liu, Z.; Streb, C.; He, H.; Yang, C.; Zhang, S.; Zhang, G. Highly Selective Electroreduction of N_2 and CO_2 to Urea over Artificial Frustrated Lewis Pairs. *Energy Environ. Sci.* **2021**, *14* (12), 6605–6615. https://doi.org/10.1039/D1EE02485J.

(29) Yuan, M.; Chen, J.; Bai, Y.; Liu, Z.; Zhang, J.; Zhao, T.; Wang, Q.; Li, S.; He, H.; Zhang, G. Unveiling Electrochemical Urea Synthesis by Co-Activation of CO_2 and N_2 with Mott–Schottky Heterostructure Catalysts. *Angew. Chem. Int. Ed.* **2021**, *60* (19), 10910–10918. https://doi.org/10.1002/anie.202101275.

(30) Yuan, M.; Chen, J.; Zhang, H.; Li, Q.; Zhou, L.; Yang, C.; Liu, R.; Liu, Z.; Zhang, S.; Zhang, G. Host–Guest Molecular Interaction Promoted Urea Electrosynthesis over a Precisely Designed Conductive Metal–Organic Framework. *Energy Environ. Sci.* **2022**, *15* (5), 2084–2095. https://doi.org/10.1039/D1EE03918K.

(31) Yuan, M.; Chen, J.; Bai, Y.; Liu, Z.; Zhang, J.; Zhao, T.; Shi, Q.; Li, S.; Wang, X.; Zhang, G. Electrochemical C–N Coupling with Perovskite Hybrids toward Efficient Urea Synthesis. *Chem. Sci.* **2021**, *12* (17), 6048–6058. https://doi.org/10.1039/D1SC01467F.

(32) Mukherjee, J.; Paul, S.; Adalder, A.; Kapse, S.; Thapa, R.; Mandal, S.; Ghorai, B.; Sarkar, S.; Ghorai, U. K. Understanding the Site-Selective Electrocatalytic Co-Reduction Mechanism for Green Urea Synthesis Using Copper Phthalocyanine Nanotubes. *Adv. Funct. Mater.* **2022**, *32* (31), 2200882. https://doi.org/10.1002/adfm.202200882.

(33) Yang, S.; Deng, J.; Chen, J.; Tan, Q.; Liu, T.; Chen, K.; Han, D.; Ma, Y.; Dai, M.; Niu, L. Photocatalytic C–N Coupling towards Urea Synthesis with a Palladium-Supported CeO_2 Catalyst. *Catal. Sci. Technol.* **2023**, *13* (6), 1855–1865. https://doi.org/10.1039/D2CY02086F.

(34) Maimaiti, H.; Xu, B.; Sun, J.; Feng, L. Photocatalytic Synthesis of Urea (CO_2/N_2/H_2O) on Coal-Based Carbon Nanotubes with the Fe-Core-Supported Ti_3^+-TiO_2 Composite Catalyst. *ACS Sustainable Chem. Eng.* **2021**, *9* (20), 6991–7002. https://doi.org/10.1021/acssuschemeng.1c00644.

(35) Wang, Y.; Wang, S.; Gan, J.; Shen, J.; Zhang, Z.; Zheng, H.; Wang, X. Photocatalytic Coreduction of N_2 and CO_2 with H_2O to $(NH_2)_2CO$ on 2D-CdS/3D-BiOBr. *ACS Sustainable Chem. Eng.* **2023**, *11* (5), 1962–1973. https://doi.org/10.1021/acssuschemeng.2c06827.
